# Bacterial Meningitis in Malawian Adults, Adolescents, and Children During the Era of Antiretroviral Scale-up and *Haemophilus influenzae* Type b Vaccination, 2000–2012

**DOI:** 10.1093/cid/ciu057

**Published:** 2014-02-04

**Authors:** Emma C. Wall, Dean B. Everett, Mavuto Mukaka, Naor Bar-Zeev, Nicholas Feasey, Andreas Jahn, Mike Moore, Joep J. van Oosterhout, Paul Pensalo, Kenneth Baguimira, Stephen B. Gordon, Elizabeth M. Molyneux, Enitan D. Carrol, Neil French, Malcolm E. Molyneux, Robert S. Heyderman

**Affiliations:** 1 Malawi-Liverpool-Wellcome Trust Clinical Research Programme, University of Malawi College of Medicine, Blantyre, Malawi; 2 The Liverpool School of Tropical Medicine; 3 Institute of Infection and Global Health, University of Liverpool, United Kingdom; 4 I-TECH Malawi; 5 Department for HIV and AIDS, Ministry of Health, Lilongwe, Malawi; 6 Department of Medicine; 7 Department of Paediatrics, University of Malawi College of Medicine, Blantyre; 8 Department of Women's and Children's Health, Institute of Translational Medicine, University of Liverpool, United Kingdom

**Keywords:** meningitis, Africa, HIV, vaccination, antiretroviral therapy

## Abstract

***Background.*** We documented bacterial meningitis trends among adults and children presenting to a large teaching hospital in Malawi during introduction of *Haemophilus influenzae* type b (Hib) vaccination and the rollout of antiretroviral therapy (ART).

***Methods.*** We analyzed data from 51 000 consecutive cerebrospinal fluid (CSF) samples obtained from adults, adolescents, and children with suspected meningitis admitted to the Queen Elizabeth Central Hospital, Blantyre, Malawi, between 2000 and 2012.

***Results.*** There was a significant decline in the total number of CSF isolates over 12 years (incident rate ratio [IRR], 0.93; 95% CI, .92–.94; *P* < .001). This decline was entirely in children aged <5 years (IRR, 0.87; 95% CI, .85–.88; *P* < .001) and coincided with the introduction of Hib vaccination. The number of adult isolates has remained unchanged (IRR, 0.99; 95% CI, .97–1.0; *P* = .135) despite rapid scale-up of ART provision. In children aged <5 years, *Streptococcus pneumoniae*, nontyphoidal salmonellae (NTS), and Hib were the most frequently isolated pathogens, and have declined over this time period. *Streptococcus pneumoniae* was the most frequently isolated pathogen in older children and adults. Estimated incidence of bacterial meningitis in 2012 was 20 per 100 000 cases in children aged <14 years, 6 per 100 000 adolescents, and 10 per 100 000 adults.

***Conclusions.*** Rates of bacterial meningitis have declined in children, but not adults, coinciding with the introduction of Hib vaccination. The highly successful rollout of ART has not yet resulted in a reduction in the incidence in adults where the burden remains high. Long-term surveillance of bacterial meningitis outside of the epidemic “meningitis belt” in Africa is essential.

## Abstract (precis)

Culture positive bacterial meningitis has fallen over a 12-year period in urban Malawi following Hib vaccination. Hib, NTS, and pneumococcal meningitis have fallen significantly in children. Pneumococcal meningitis has not fallen in adults; NTS and pneumococcal meningitis are seasonal.

The burden of acute bacterial meningitis (ABM) across sub-Saharan Africa is disproportionally higher than in countries in the developed world [1]. The associated mortality is more than twice that of developed countries for both children (25%–36% vs 5%–11%) and adults (54%–70% vs 25%–30%) [2–5]. Outside the “meningitis belt” where large epidemics of meningococcal meningitis occur every 5–10 years, bacterial meningitis incidence in children in Africa is estimated to be as high as 25 cases per 100 000 children [6], compared with 0.56 per 100 000 in children aged 2–10 years in the United States or 1–3 per 100 000 in the United Kingdom [7–9]. The incidence in well-resourced countries continues to decline with the introduction of meningococcal and pneumococcal vaccines [10]. Estimates of meningitis incidence in African adults are lacking, but a higher burden of disease is likely, compared with resource-rich countries, particularly where human immunodeficiency virus (HIV) is highly prevalent [5]. In Malawi, where the population prevalence of HIV is estimated to be 11.9% [11], inpatient HIV prevalence is 70% in adults, and 85% in adults with bacterial meningitis [12]. Of children with ABM in Malawi, 36% are HIV infected [13].

In the last 10 years, numerous public health interventions have been introduced into Malawi, including *Haemophilus influenzae* type b (Hib) vaccination in 2002 [14], intensification of malaria control in 2005 [15], rapid scale-up of antiretroviral therapy (ART) in 2004, and co-trimoxazole preventive therapy in 2005 [16]. These measures have been associated with considerable improvements in under-5 childhood mortality and decreases in the mortality rate of HIV-infected adults from 42% to 17% [17, 18]. However, it is less certain how these interventions have affected the burden of ABM. We have been conducting surveillance for invasive bacterial infections for almost 15 years at the largest district and referral hospital in Malawi. We have taken advantage of these consistently collected data to analyze trends in etiology and disease incidence for ABM over a 12-year period.

## METHODS

### Setting

The Queen Elizabeth Central Hospital (QECH) in Blantyre, Malawi, is a 1250-bed government-funded teaching hospital providing free healthcare that serves approximately 1 million people. The annual admission rate to QECH is 50 000 per year. The diagnostic laboratory at the Malawi-Liverpool-Wellcome Trust (MLW) Clinical Research Programme has provided a routine cerebrospinal fluid (CSF) culture for clinically suspected meningitis at QECH for >10 years.

### Patients

Since 2000, all adults (aged ≥20 years) and children and adolescents presenting with a clinical suspicion of meningitis routinely underwent lumbar puncture (LP): 5–10 mL of CSF from adults and adolescents, and 1–2 mL from children. LP was withheld in patients with clear contraindications to the procedure [19], but this was rare. Indications for LP did not change during the study period. Culture-positive isolates were divided into clinically relevant age groups: neonates (0 to <3 months of age), Expanded Program on Immunization (EPI)–eligible children (aged 3 months to <5 years), older children (aged 5–14 years), adolescents (aged 15–19 years), and adults (aged ≥20 years). Neonatal data were not analyzed as a separate group in this study as neonatal meningitis is epidemiologically distinct from community-acquired meningitis. However, these children were included in the overall trend analysis due to a small number of isolates having no patient age assigned. This study conforms to institutional guidelines and practices. Data collection was approved by the University of Malawi College of Medicine Research Ethics Committee.

### Pathogen Isolation and Identification

Gram stain was performed if the CSF white cell count (WCC) was >10 cells/µL. India ink stain was performed on adult CSF samples. All samples were cultured on sheep blood and chocolate agar for 48 hours under aerobic and microaerophilic conditions. Bacteria and fungi were identified using standard methods [20]. Antibiotic susceptibilities of all bacterial isolates were determined by the disc diffusion method (Oxoid, United Kingdom) using standard guidelines [21]. CSF biochemistry was determined using a Beckman Coulter CX5 Synchron Pro analyzer from 2009 onward.

Cryptococcal culture rates on blood agar were reported. Mycobacterial CSF culture was not performed. All diagnostic testing at the MLW Clinical Research Programme laboratory was quality controlled as part of internationally recognized quality control programs.

### National HIV Intervention Strategies

In Blantyre, ART rollout began from QECH to health centers in 2004. ART Scale-up data were obtained from the Malawi Ministry of Health [22]. Data on co-trimoxazole preventive therapy usage were not available.

### Rainfall and Temperature

Rainfall and temperature data were obtained from the Department of Climate Change and Meteorological Services, Malawi, for 2 stations in Blantyre for 2000–2010. The cooler dry season is between May and August, the hot dry season between September and November, and the rainy season between November and April.

### Incidence Rates

Midyear population estimates were based on projections from the 1998 and 2008 Population and Housing Censuses [23]. Incidence was defined as cases number in a defined period divided by population time observed. Changes in incidence over time were plotted.

### Time-Series Analysis

Time-series decomposition was used to separate long-term trends from seasonal perturbations [24]. Symmetric locally weighted moving averages were used, with less weight given to time points (in months) further from the present. A 5-month window was used to detect seasonality:

$$\hat Y_t = \displaystyle{1 \over 9}(Y_{t - 2} + 2Y_{t - 1} + 3Y_t + 2Y_{t + 1} + Y_{t + 2} ).$$

A 12-month window was used to construct a trend line that would be sensitive to year-to-year changes but dampen noise from seasonal perturbation:

$$\eqalign{\widehat{Y}_t = & \displaystyle{1 \over {24}}(Y_{t - 6} + Y_{t + 6} ) + \displaystyle{1 \over {12}}(Y_t + Y_{t - 1} + Y_{t + 1} + Y_{t - 2} + Y_{t + 2} \cr &+ Y_{t - 3} + Y_{t + 3} + Y_{t - 4} + Y_{t + 4} + Y_{t - 5} + Y_{t + 5}). } $$

To determine whether seasonal perturbation differed significantly from baseline, data were trend differenced. The distribution of residual fluctuations for each month was compared to baseline with 2-tailed *t* test. To determine the impact of rainfall and temperature, an autoregressive integrated moving average model with exogenous variables (ARMAX) model was used. Models using specifications of the autoregressive and moving average parameters were compared using the absolute percentage error.

### Statistical Methods

The Kruskal-Wallis test was used to detect globally significant differences in the distribution of nonnormal variables among groups of interest. Where results were significant, pairwise comparisons were done using Mann-Whitney test. Poisson regression was used to derive incident rate ratios (IRRs) and 95% confidence intervals (CIs) over the 12-year period. Disease incidence ratios pre- and postvaccination were derived using binary (post–pre) rate ratios to estimate vaccine efficacy. A *P* value <.05 denoted statistical significance. Statistical and time series analyses were done using Stata software, version 12.1 (StataCorp, College Station, Texas).

## RESULTS

### Trends in Disease and Pathogen Incidence Over Time

**Figure 1.**
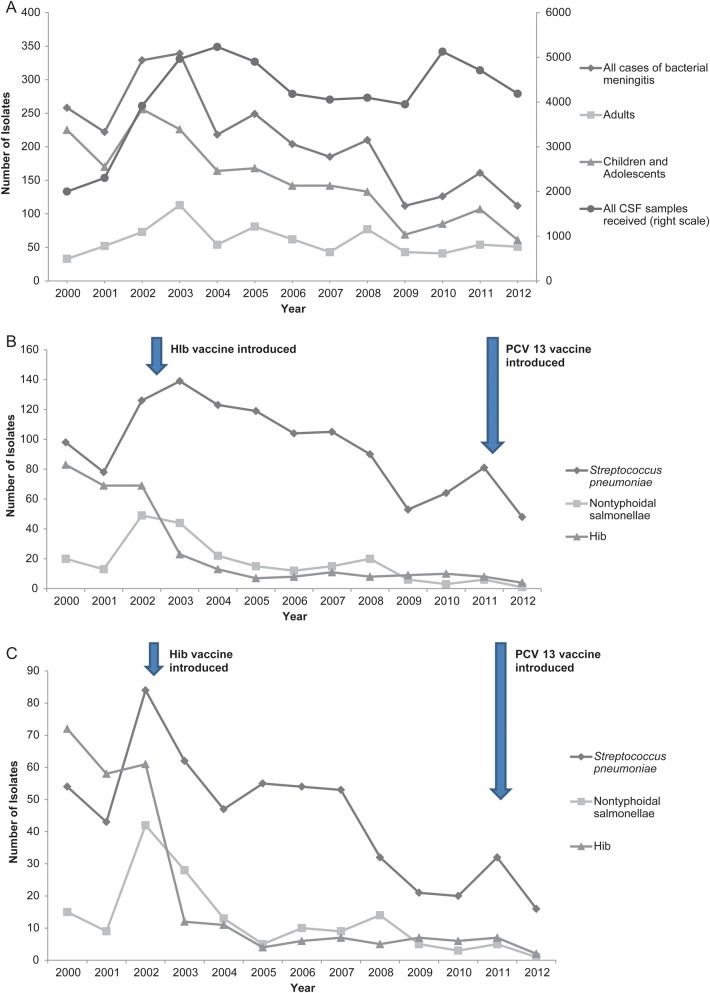
Trends in culture positive isolates over time. *A*, Total numbers of culture-positive cases of bacterial meningitis, 2000–2012, by year. Isolates are subdivided into adults aged >20 years and children and adolescents aged <20 years, compared with all cerebrospinal fluid samples received by the laboratory for analysis (right scale). *B*, Total numbers of culture-positive isolates by year and pathogen in all children and adolescents aged <20 years of *Streptococcus pneumoniae*, nontyphoidal salmonellae (NTS), and *Haemophillus influenzae* (Hib) meningitis. *C*, Total numbers of culture-positive isolates by year and pathogen in age-known Expanded Program on Immunization vaccine-eligible children aged ≥3 months to <5 years of *S. pneumoniae*, NTS, and Hib meningitis. Abbreviations: CSF, cerebrospinal fluid; Hib, *Haemophilus influenzae*; PCV13, 13-valent pneumococcal conjugate vaccine.

The overall trends in all culture-positive cases of bacterial meningitis in adults, adolescents, and children aged <20 years are described in Figure 1*A*. There was a significant annual decline in the total number of CSF isolates between 2000 and 2012 (IRR, 0.93; 95% CI, .92–.94; *P* < .001). This decline was entirely in children and adolescents (IRR, 0.87; 95% CI, .85–.88; *P* < .001); the number of isolates from adults remained unchanged (IRR, 0.99; 95% CI, .97–1.0; *P* = .135).The number of CSF samples received by the laboratory each year remained constant after 2002, and hospital admission rates did not change during the study period, despite an increasing population.

Specific trends in the most commonly isolated pathogens in children and adolescents are described in Figure 1*B*. These data include all children from birth where the age is known, and a small number of children (<5% total) where the age was not documented but admission to a pediatric ward was recorded. Cases of Hib meningitis declined 20-fold from 80 cases per year in 2000 to <4 cases per year in 2012 in all children and adolescents. Following Hib vaccination in 2002, cases declined rapidly in the EPI-eligible, 3 months to <5 years age group (Figure 1*C*). In the years immediately preceding and following vaccine introduction, Hib incidence has dropped dramatically; the binary IRR of the fall is 0.11 (95% CI, .08–.14; *P* < .001), providing an estimated vaccine effectiveness of 89% (95% CI, 86%–92%). Childhood nontyphoidal salmonellae (NTS) and pneumococcal meningitis cases have also significantly declined in children in this age group between 2002 and 2012; the IRR over 12 years was 0.85 (95% CI, .81–.89; *P* < .001) for NTS, and 0.88 (95% CI, .85–.91; *P* < .001) for pneumococcus.

**Figure 2.**
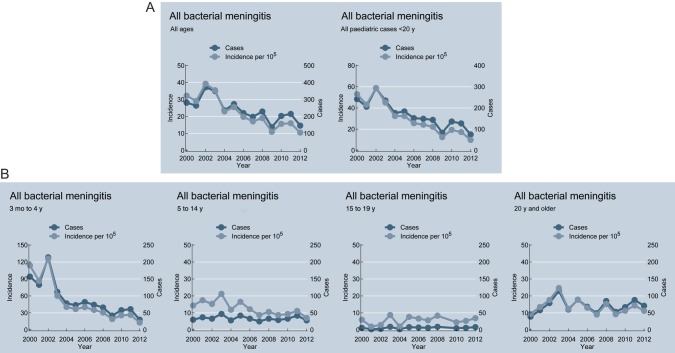
Incidence of culture-positive bacterial meningitis per 100 000 population. *A*, Incidence and all cases of bacterial meningitis, 2000–2012, in all cases and children and adolescents aged <20 years. *B*, Incidence and cases by specific age group (only children for whom age was known are included).

From these data, the overall incidence of culture-positive bacterial meningitis was estimated. Incidence declined from 49.6 per 100 000 in 2002 to 20 per 100 000 in 2012. This decline is driven predominately by the 3 months to <5 years age group (incidence 154.4 in 2002 to 20 in 2012), and the 5-15 years group (incidence 15.7 in 2002 to 8 in 2012), with incidence in adolescents and adults remaining unchanged (Figure 2).

Supplementary Figure 1*A*–*D* shows the proportions of all culture-positive isolates per age group. In the age group 3 months to <5 years (Supplementary Figure 1*A*), there has been a decline of 88% in the total number of microbiologically confirmed pathogens since 2000. Hib meningitis has dramatically declined from >35% of all cases prior to 2002 to 3.5% in 2005, subsequently remaining between 10% and 15%. *Streptococcus pneumoniae* accounted for 30%–50% of cases in 2000–2003, but as Hib cases declined, pneumococcus has consistently accounted for >60% of cases since 2004. The number of pneumococcal isolates in this age group has dropped from a peak of 115 out of 177 cases in 2007 to 16 per 25 cases in 2012. Nontyphoidal salmonellae meningitis caused approximately 23% of cases before 2004, dropping to <10% by 2012. Group B streptococcal meningitis peaked in 2005 at almost 15%, but since 2009 has dropped to <3%. Confirmed meningococcal meningitis has remained consistently at <5%. Pathogens listed as “other” have increased in proportion as the other causes have decreased. This group includes groups A and D streptococci, *Klebsiella pneumoniae*, *Listeria monocytogenes*, *Salmonella enteritidis*, *Staphylococcus aureus*, and *H. influenzae* types a and c and nontypeables.

**Figure 3.**
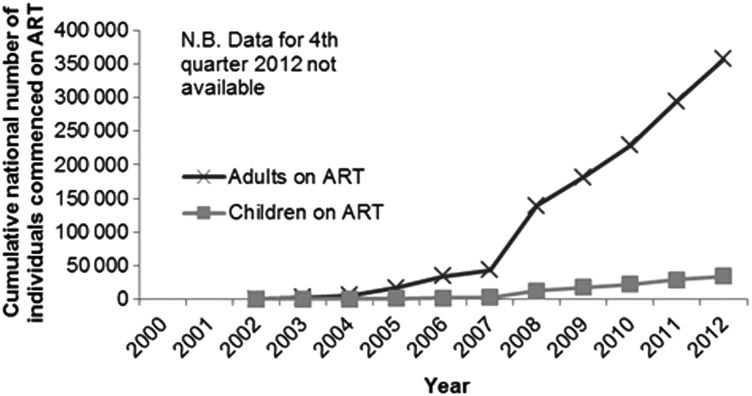
Cumulative numbers of adults and children alive on antiretroviral therapy (ART) per year in the Southern Region of Malawi.

*Streptococcus pneumoniae* is the predominant bacterial pathogen causing 65% of infections in the 5- to 15-year age group; Hib caused 8.6% and NTS <1% of infections (Supplementary Figure 1*B*). *Streptococcus pneumoniae* was also the predominant pathogen in all older age groups >15 years; numbers of *Neisseria meningitidis* isolated from adults and adolescents were small (Supplementary Figure 1*C* and 1*D*). Rapid up-scaling of ART has led to >354 000 adults and >34 000 children starting ART by September 2012 in the Southern Region of Malawi (Figure 3), of which >215 000 adults and >21 000 children were reported to be alive on ART at the end of 2012. However meningitis due to *Cryptococcus neoformans* accounted for significant and consistent numbers of cases, particularly in adults in 2000–2012, with the exception of the under-5 group, in whom cryptococcal meningitis was detected only from 2009 onward (Supplementary Figure 2).

### Seasonality

**Figure 4.**
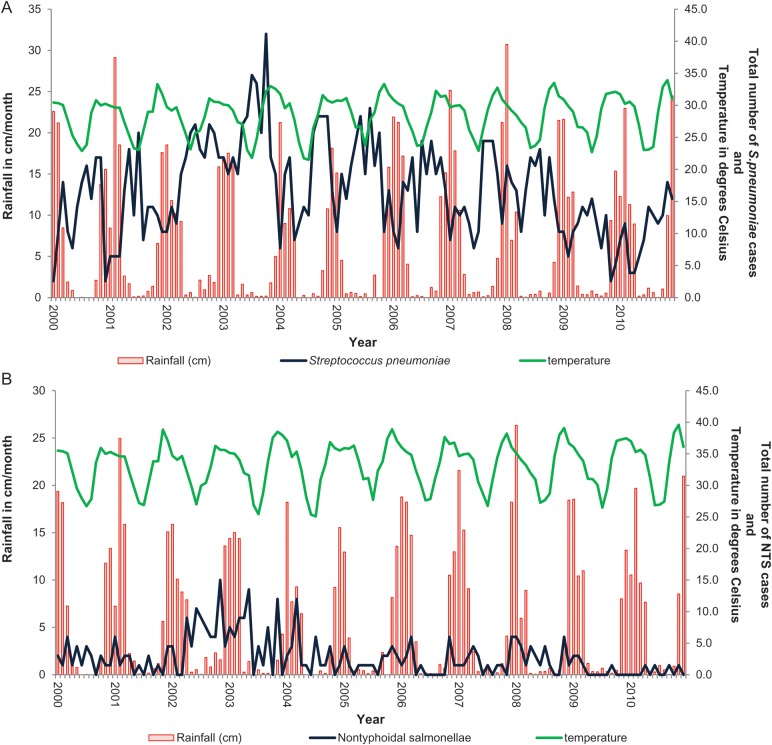
Seasonality plot of all meningitis cases caused by *Streptococcus pneumoniae* (*A*) and nontyphoidal salmonellae (NTS) (*B*). *A*, Seasonality of *S. pneumoniae. B*, Seasonality of NTS.

We have previously described seasonal invasive pneumococcal disease trends in Blantyre over the same time period [25] and tested if bacterial meningitis is seasonal. Figure 4*A* and 4*B* describe the seasonality of meningitis due to *S. pneumoniae* and *Salmonella* Typhimurium. Rainfall and temperature data were available for the 2000–2010 period only.

The mean number of isolates per month of *S. pneumoniae* were significantly and consistently lower than the baseline long-term trend during the wet season between December to April over 10 years, despite the overall fall in isolate numbers (*P* < .05). Using the ARMAX model, rainfall was associated with 28% (95% CI, 13.7%–41.4%; *P* < .001) fewer cases of pneumococcal meningitis. Hot weather was associated with 42.8% (95% CI, 1.8%–87.5%, *P* = .06) more cases of pneumococcal meningitis, but the confidence interval crosses the null point. *Streptococcus pneumoniae* meningitis is therefore a seasonal disease in Malawi occurring in the dry season, particularly hot, dry months. Cases of NTS meningitis were above the mean in August to December and below the mean in January, but the trend did not reach significance. Hib and cryptococcal disease were not seasonal (data not shown).

### CSF Findings by Pathogen

Significantly lower CSF WCCs were seen in cases of *S. pneumoniae* meningitis compared with *N. meningitidis* meningitis in both adults and children (Table 1). HIV did not influence the CSF WCC in pneumococcal meningitis in adults (median CSF WCC in HIV-positive adults was 435 cells/µL [interquartile range {IQR}, 107–1680 cells/µL]; n = 297), compared with 575 cells/µL (IQR, 196–1740 cells/µL) in HIV-negative adults (n = 44) (*P* = .31). Both NTS and cryptococcal meningitis were associated with lower CSF WCC than *S. pneumoniae* and *N. meningitidis.* CSF protein was significantly higher in bacterial meningitis caused by all pathogens (with the exception of NTS) than in cryptococcal meningitis (*P* < .001). Similarly, CSF glucose levels were significantly lower in *S. pneumoniae* compared with cryptococcus for both adults and children (*P* < .001).

**Table 1.** Differences Between Cerebrospinal Fluid (CSF) Laboratory Parameters From Culture-Positive CSF Samples, by Infecting Organism and Age Group

CSF Parameter	*Spn* Meningitis, Adults (n = 795)	*Spn* Meningitis, Children (n = 998)	NTS Meningitis, Adults (n = 28)	NTS Meningitis, Children (n = 269)	NM Meningitis, Adults (n = 17)	NM Meningitis, Children (n = 22)
Median CSF protein, g/L (IQR)	2.6 (0–4.6) (n = 161)	2.4 (0.99–3.59) (n = 170)	1.84 (0.92–3.15) (n = 3)	1.84 (0–5.31) (n = 26)	4.53 (3.23–5.54) (n = 4)	1.87 (1.58–3.20) (n = 4)
Median CSF glucose, mmol/L (IQR)	0 (0–0.17) (n = 161)	0 (0–0.33) (n = 146)	2.75 (0–3.08) (n = 3)	0.06 (0–0.44) (n = 25)	0.08 (0–0.98) (n = 4)	0.42 (0.14–0.73) (n = 4)
Median CSF WCC, cells/µL (IQR)	335 (74–1360)	407 (130–1360)	65 (5–700)	560 (150–2800)	2320 (155–5920)	1970 (250–6800)

Adults are defined as aged >20 years, children as children and adolescents aged <20 years.

Abbreviations: CSF, cerebrospinal fluid; IQR, interquartile range; NM, *Neisseria meningitidis*; NTS, nontyphoidal salmonellae; *Spn*, *Streptococcus pneumoniae*; WCC, white cell count*.*

WCC data were collected for culture-negative CSF specimens from 2007. Between 2007 and 2010, 643 of 8166 (7.8%) adults had a negative culture but had a CSF WCC of >100 cells/µL; 796 of 9431 (8.4%) children had a negative culture but had a CSF WCC of >20 cells/µL. These cases may represent a significant additional meningitis disease burden; we are currently investigating the causes of culture-negative meningitis in our hospital.

## DISCUSSION

Our data indicate that culture-confirmed cases of ABM presenting to the largest secondary and tertiary referral hospital in Malawi have declined steeply over the last 12 years, driven largely by a decline in persons aged 3 months to <5 years. This has occurred prior to the addition of the 13-valent pneumococcal conjugate vaccine (PCV13) to the routine infant schedule in Malawi in 2011 but subsequent to the introduction of Hib vaccine in 2002. Although a similar decline in Hib ABM that was ecologically coincident with Hib vaccine introduction has been observed elsewhere in Africa [26, 27], it is striking that we also observed a decline in cases of pneumococcal and NTS ABM in this age group. Why we have not observed a similar overall decline in the incidence of acute adult ABM, in contrast to childhood disease, is uncertain.

The decline in childhood cases of *S. pneumoniae* ABM in children aged 3 months to <5 years preceding the introduction of PCV13 has not been observed in other sub-Saharan African facility-based studies [28], with the exception of HIV-infected children receiving ART in South Africa [29]. We have previously reported a similar decline in cases of pneumococcal bacteremia (IPD) in adults and children [25]. During 2000–20012 in Malawi, there have been considerable improvements in general nutrition and the management of childhood illness at primary healthcare levels, which have been linked to a 50% decline in the overall under-5 mortality between 1999 and 2009 [30]. Pediatric ABM has been associated with HIV in 36% of cases in Malawi [13]. Data on the overall community prevalence of HIV in children are unavailable, but the estimated prevalence of HIV in pregnant women in Southern Malawi has fallen from 19% in 2000 to 14% in 2010 [22]. Furthermore, extensive rollout of ART for children and a national prevention of mother-to-child transmission program may be driving down the burden of HIV. We suggest that these factors, together with some improvement in socioeconomic indicators [31], are likely to have contributed to the observed decline in childhood ABM, particularly NTS and *S. pneumoniae* meningitis. Although the control of malaria has been shown to improve all-cause mortality in other African settings [32], there has not been a sufficiently large decline in childhood malaria in Malawi to contribute substantially to this trend [15].

Free ART has been provided in Malawi for adults since 2004, and to date nearly 360 000 HIV-infected individuals had been registered in the national ART program in the Southern Region of Malawi; co-trimoxazole prophylaxis has been widely available since 2006 [22].The absence of a decline in adult ABM, despite improvements in HIV care including the rollout of ART and co-trimoxazole, coincident with a fall in the incidence of IPD, is therefore surprising [25]. We have previously demonstrated high rates of pneumococcal carriage with broad serotype diversity in HIV-infected adults despite the initiation of ART [33]. We therefore speculate that persistent defects in immune control in the nasopharynx and potentially the CSF compartment could account for the continuing high incidence of pneumococcal ABM in adults.

Meningitis is seasonal in many parts of the world [34]. A better understanding of these disease patterns and the potential climatic associations are important for public health planning and the interpretation of disease incidence. Malawi is geographically far from countries where epidemic meningitis primarily due to *N. meningitidis,* coincident with the hot, dry season has been described [34, 35]. In Malawi, pneumococcal and NTS meningitis were both seasonal over the last 10 years, peaking in the cold, dry and hot, wet seasons, respectively. Importantly, seasonality was maintained, even as incidence rates have declined. Other African centers beyond the meningitis belt have observed meningitis seasonality in Uganda, but not in Tanzania, although these data are not from centers undertaking long-term surveillance [36, 37].

Our disease estimates are of minimum incidence, as it is well described that routine surveillance supported by CSF culture alone may underestimate meningitis incidence [38]. However, in a high-HIV-prevalence setting, the causes of culture-negative meningitis includes tuberculosis. We did not have access to tuberculosis culture, so incidence estimates including culture-negative cases may be inaccurate and are not presented.

Our data have several limitations. Several adult and pediatric ABM and IPD clinical trials conducted at our center may have increased case ascertainment. However, following the completion of these studies, the number of CSF samples processed in the laboratory has remained static. There may have been underascertainment of pathogens due to the unavailability of diagnostic nucleic acid detection [38]. Although there are no other government-funded facilities where ABM cases are routinely admitted, patients may experience significant delays in the community and die undiagnosed, causing further underascertainment [39]. Health centers may have administered parenteral antibiotics prior to transfer to QECH, but documentation and trends are not available. There were no changes in either national or hospital guidelines during this period. Finally, our data set has arisen from 1 center. However, given the similarity of cross-sectional data that have emerged with other regional settings, it is likely that these data are generalizable to other HIV-prevalent African countries.

In conclusion, rates of ABM have declined significantly in children but not in adults in Malawi. Hib vaccine introduction appears to have resulted in the decline of incidence of Hib in young children, but the highly successful rollout of ART has not yet resulted in a reduction in the incidence in adults, among whom the burden remains high. Long-term surveillance of bacterial meningitis outside the epidemic meningitis belt is essential in Africa.

## Supplementary Data

Supplementary materials are available at *Clinical Infectious Diseases* online (http://cid.oxfordjournals.org). Supplementary materials consist of data provided by the author that are published to benefit the reader. The posted materials are not copyedited. The contents of all supplementary data are the sole responsibility of the authors. Questions or messages regarding errors should be addressed to the author.

Supplementary Data
