# Supplementary material for: Bacterial Meningitis in Malawian Adults, Adolescents, and Children During the Era of Antiretroviral Scale-up and Haemophilus influenzae Type b Vaccination, 2000–2012
Source: Clin Infect Dis. 2014 Feb 4;58(10):e137–45. doi: 10.1093/cid/ciu057 (PMC4001285; doi:10.1093/cid/ciu057)
Supplement: Supplementary Data [file supp_58_10_e137__index.html]

Bacterial Meningitis in Malawian Adults, Adolescents, and Children During the Era of Antiretroviral Scale-up and Haemophilus influenzae Type b Vaccination, 2000–2012 — Bacterial Meningitis in Malawian Adults, Adolescents, and Children During the Era of Antiretroviral Scale-up and Haemophilus influenzae Type b Vaccination, 2000–2012 — Supplementary Data 

# Bacterial Meningitis in Malawian Adults, Adolescents, and Children During the Era of Antiretroviral Scale-up and *Haemophilus influenzae* Type b Vaccination, 2000–2012

## Supplementary Data

Supplementary Data

**Files in this Data Supplement:**

- Supplementary Data - Docx file
