# Supplementary material for: Bacterial Meningitis in Malawian Adults, Adolescents, and Children During the Era of Antiretroviral Scale-up and Haemophilus influenzae Type b Vaccination, 2000–2012
Source: Clin Infect Dis. 2014 Feb 4;58(10):e137–45. doi: 10.1093/cid/ciu057 (PMC4001285; doi:10.1093/cid/ciu057)

**Supplementary Figure 1**: Frequently isolated individual pathogens as percentages of all culture positive isolates per year by age group (1a-d). Age-known only children included.

**Supplementary Figure 1a**: EPI eligible children aged 3 months to less than 5 years

**Supplementary Figure 1b**: 5 years - 15 years

**Supplementary Figure 1c**: Adolescents aged 15-19 years

**Supplementary Figure 1d**: Adults 20 years and older

**Supplementary Figure 2**: Proportions of all culture positive meningitis caused by *Cryptococcus neoformans* compared to all cause bacterial meningitis by age group.


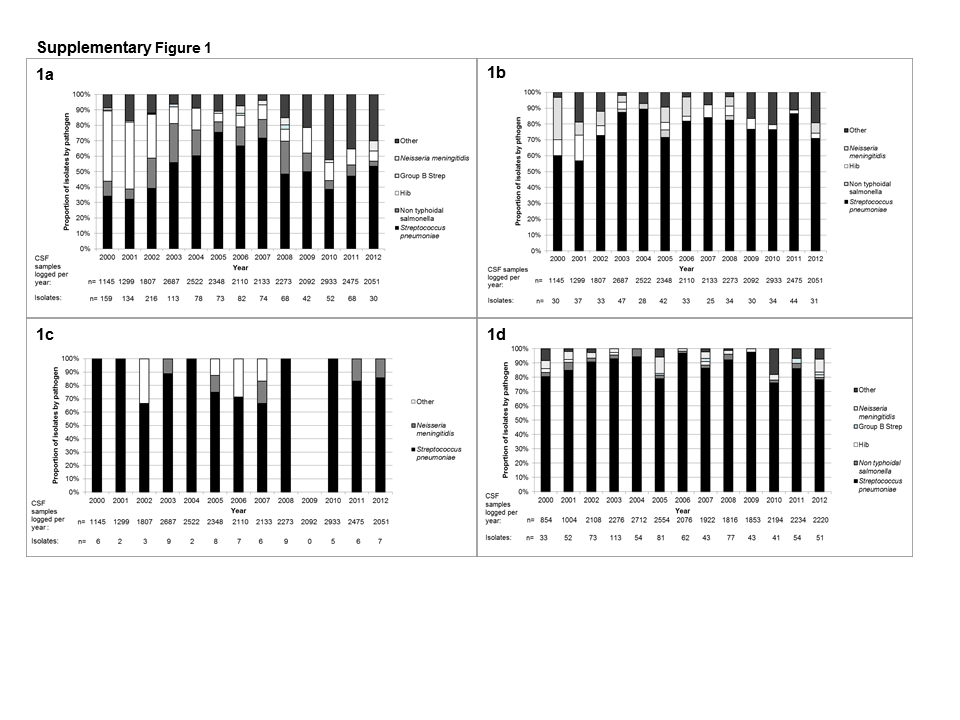

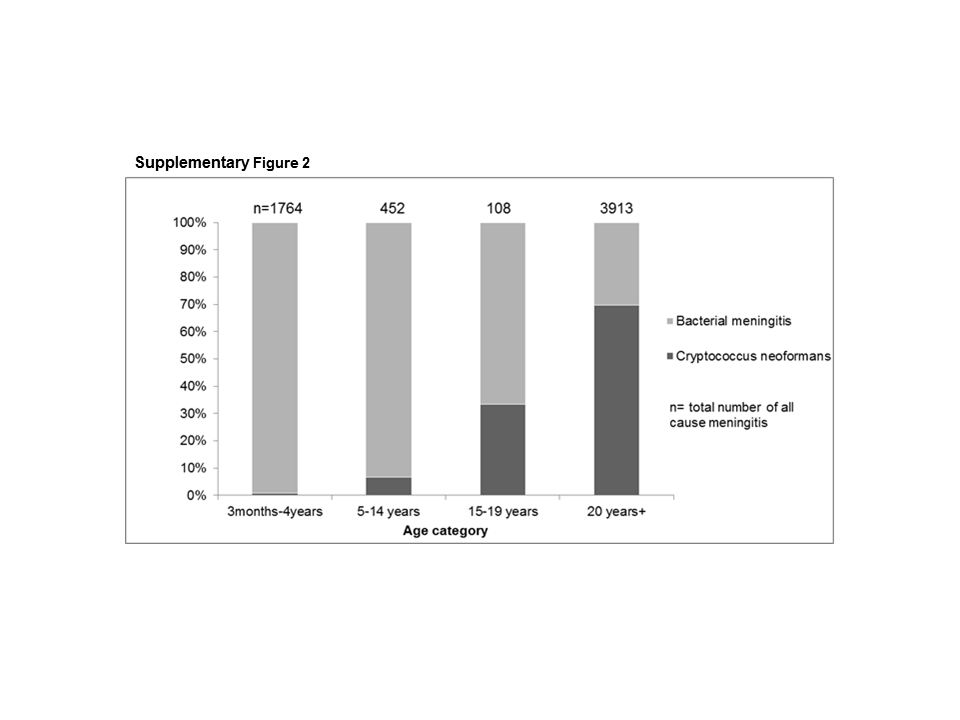

Supplement: Supplementary Data [file supp_ciu057_ciu057supp.docx]
